# Practitioner Review: What have we learnt about the causes of ADHD?

**DOI:** 10.1111/j.1469-7610.2012.02611.x

**Published:** 2013-01

**Authors:** Anita Thapar, Miriam Cooper, Olga Eyre, Kate Langley

**Affiliations:** 1 Child & Adolescent Psychiatry Section, Institute of Psychological Medicine and Clinical Neurosciences, Cardiff University School of MedicineCardiff; 2 MRC Centre for Neuropsychiatric Genetics and Genomics, Cardiff University School of MedicineCardiff, UK

**Keywords:** ADHD, genetics, risk factors, perinatal, prenatal, aetiology, environmental–gene interactions

## Abstract

**Background:**

Attention deficit hyperactivity disorder (ADHD) and its possible causes still attract controversy. Genes, pre and perinatal risks, psychosocial factors and environmental toxins have all been considered as potential risk factors.

**Method:**

This review (focussing on literature published since 1997, selected from a search of PubMed) critically considers putative risk factors with a focus on genetics and selected environmental risks, examines their relationships with ADHD and discusses the likelihood that these risks are causal as well as some of the main implications.

**Results:**

No single risk factor explains ADHD. Both inherited and noninherited factors contribute and their effects are interdependent. ADHD is familial and heritable. Research into the inherited and molecular genetic contributions to ADHD suggest an important overlap with other neurodevelopmental problems, notably, autism spectrum disorders. Having a biological relative with ADHD, large, rare copy number variants, some small effect size candidate gene variants, extreme early adversity, pre and postnatal exposure to lead and low birth weight/prematurity have been most consistently found as risk factors, but none are yet known to be definitely causal. There is a large literature documenting associations between ADHD and a wide variety of putative environmental risks that can, at present, only be regarded as correlates. Findings from research designs that go beyond simply testing for association are beginning to contest the robustness of some environmental exposures previously thought to be ADHD risk factors.

**Conclusions:**

The genetic risks implicated in ADHD generally tend to have small effect sizes or be rare and often increase risk of many other types of psychopathology. Thus, they cannot be used for prediction, genetic testing or diagnostic purposes beyond what is predicted by a family history. There is a need to consider the possibility of parents and siblings being similarly affected and how this might impact on engagement with families, influence interventions and require integration with adult services. Genetic contributions to disorder do not necessarily mean that medications are the treatment of choice. We also consider how findings might influence the conceptualisation of ADHD, public health policy implications and why it is unhelpful and incorrect to dichotomise genetic/biological and environmental explanations. It is essential that practitioners can interpret genetic and aetiological research findings and impart informed explanations to families.

## Introduction

Despite still periodically attracting public controversy, attention deficit hyperactivity disorder (ADHD) has long been recognised as a clinical entity (Taylor, 2011). In the last few decades, there has been an enormous amount of research into the aetiology of ADHD. What should we believe? How do research findings impact on clinical practice if at all? In this article, our aims are to critically review selected factors that have been considered as likely contributors to ADHD risk, update practitioners on key findings and their interpretation and provide some ‘take-home’ messages. In accordance with Kraemer et al. (Kraemer, Stice, Kazdin, Offord, & Kupfer, 2001), we use the terms ‘risk’ to mean the probability of developing an outcome (herein, ADHD) within a population of subjects, ‘correlate’ where a factor is associated with outcome, ‘risk factor’ where a factor precedes outcome and ‘causal risk factor’ where a factor alters the risk of outcome when manipulated.

### Methods

This is a narrative review, focusing on literature in the last 15 years, selected from a PubMed search. The key words used were ADHD and genetic, inherited, gene-environment interaction, gene-environment interplay, aetiology, linkage, genome-wide association study, copy number variant, twin studies, adoption studies, COMT, conduct disorder, candidate gene, environment, toxins, lead, polychlorinated biphenyls, PCB, pesticides, diet, prenatal, perinatal, pregnancy, smoking, tobacco, substance, stress, birth weight and prematurity. We supplemented these with reviews and book chapters and emphasise replicated findings.

## An inherited contribution to ADHD

Attention deficit hyperactivity disorder, like other psychiatric and developmental disorders runs in families. Genetic risk when passed on from parents is known as inherited risk/liability; not all genetic risks are necessarily inherited (see later). First degree relatives of those with ADHD are two to eight times more likely than relatives of unaffected individuals to also show ADHD (Faraone et al., 2005). Twin studies in many different countries show high heritability rates for ADHD of around 71–90% (Faraone et al., 2005; Nikolas & Burt, 2010; Thapar, Holmes, Poulton, & Harrington, 1999) with evidence of shared familial/inherited risks for combined and inattentive type symptoms (Willcutt et al., 2012). Heritability estimates not only include genetic influences, but also the effects of gene-environment interplay that are very likely to be important (see later). Thus, high heritability estimates do not rule out the contribution of environmental risks. Although caution is needed in interpreting absolute estimates of heritability, what is interesting is that findings are consistent across different populations and measures. That is not always the case. For example, twin studies of child and adolescent depression show widely differing heritability estimates (Thapar & Rice, 2006). Estimates will also depend on the population studied and environmental context. For example, theoretically if everyone in a population was equally exposed to an environmental risk factor of major effect size, most variation in disorder would be explained by genetic differences. Thus, the consistency in heritability estimates is surprising as it might be expected that environmental risks would differ across time and for different countries.

Adoption studies allow separation of inherited and postnatal environmental effects by examining the degree of concordance or similarity between individuals who have ADHD and their biologically related and unrelated relatives. All five published adoption studies of ADHD (Alberts-Corush, Firestone, & Goodman, 1986; Cantwell, 1975; Cunningham, Cadoret, Loftus, & Edwards, 1975; Morrison & Stewart, 1973; Sprich, Biederman, Crawford, Mundy, & Faraone, 2000) are consistent in showing a strong inherited contribution. Do genetically informative designs like adoption studies simply show this consistent pattern of strong inherited effects for all psychopathology? The answer is that findings are not identical across all phenotypes. For example, genetically informative studies of depression and antisocial behaviour (Harold et al., 2011; Lewis, Rice, Harold, Collishaw, & Thapar, 2011; Silberg, Maes, & Eaves, 2012; Tully, Iacono, & McGue, 2008) show the important contribution of noninherited factors to intergenerational transmission of these problems and highlight that the manifestation of genetic liability varies with the environmental context (Rhee & Waldman, 2002). Although when taken with family and twin study findings, adoption studies of ADHD suggest an important inherited contribution to ADHD, they do not remove the influence of prenatal risks or early postnatal adversity. Future adoption studies of ADHD will be invaluable, especially those that involve adoption at birth and look more closely at environmental influences that modify the clinical presentation and outcomes in those at higher genetic risk.

**Figure 1:**
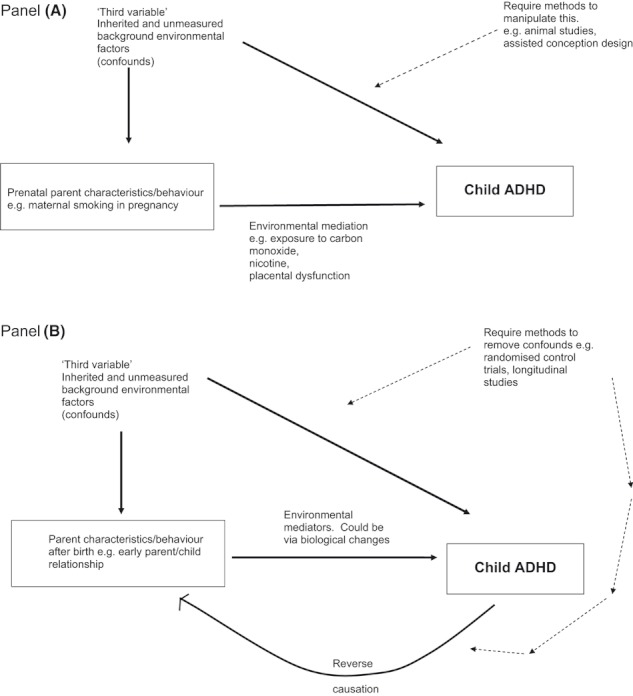
The associations of environmental risks with child attention deficit hyperactivity disorder, illustrating how environmental mediation may operate for (A) prenatal and (B) perinatal risks

Finally, as we will keep emphasising, the issue is never genes or biology versus environment given that there is overwhelming evidence that their effects are so closely intertwined (Rutter, 2006). The most important environmental risk factors for psychopathology, including ADHD and its secondary adverse consequences, are likely to be affected by genetically influenced parental and child dispositions (Rutter, 2006); for example, prenatal exposure to maternal cigarette smoking or peer rejection (gene-environment correlation). Thus, associations of environmental risks with ADHD might arise completely or partly through inherited confounds and genetic risks might operate on manifest phenotypes through environmental mechanisms (see Figure 1). Genetic risks can also influence susceptibility by altering individual sensitivity to environmental risks or protective factors (gene-environment interaction; Nigg, Nikolas, & Burt, 2010).

## The overlap of ADHD with other developmental and psychiatric disorders

ADHD shows comorbidity with a wide variety of problems (Taylor, 2011; Willcutt et al., 2012). There are well-established strong associations with lower IQ and intellectual disability, specific learning and developmental problems, such as reading disability, speech and language problems, motor co-ordination difficulties and also autistic spectrum disorders (ASDs). Recent twin studies suggest that shared inherited factors contribute to the comorbidity of ADHD and other neurodevelopmental problems, including ASDs (Lichtenstein, Carlstrom, Rastam, Gillberg, & Anckarsater, 2010; Ronald, Larsson, Anckarsater, & Lichtenstein, 2011; Ronald, Simonoff, Kuntsi, Asherson, & Plomin, 2008) and reading problems, especially with regards to the inattentive features of ADHD (Willcutt, Pennington, & DeFries, 2000). The close clinical relationships, shared genetic risk factors, coupled with other shared features, notably an early age of onset and male excess, provide a strong argument for considering ADHD as one of a group of neurodevelopmental disorders (Rutter, 2011).

However, ADHD also shows a high level of comorbidity with other psychiatric and behavioural disorders, notably conduct problems/antisocial behaviour, alcohol and substance misuse and mood disorders, that is explained by shared heritability (Cole, Ball, Martin, Scourfield, & McGuffin, 2009; Thapar, Harrington, & McGuffin, 2001; Willcutt et al., 2012). Thus, there is also a view that ADHD might be best considered as one of a cluster of externalising disorders (Andrews, Pine, Hobbs, Anderson, & Sunderland, 2009). However, one key difference from the overlap with neurodevelopmental problems is that some of these problems/disorders can present later in development and might arise as a consequence of having ADHD (analogous to diabetes potentially resulting in blindness; Fergusson & Horwood, 1995; Taylor, Chadwick, Heptinstall, & Danckaerts, 1996), although that is not necessarily the case for early onset conduct problems that are closely associated with ADHD (Willcutt et al., 2012).

## ADHD genetics

### Identifying ADHD ‘risk genes’

Genetics is not much different from other aetiological research, in that it involves testing for a relationship between variation in a risk/predictor measure and an outcome. New technologies are, however, allowing for the more detailed capture of genetic variation. For common, complex disorders, such as ADHD, the predominant starting assumption has been that risk is mainly explained by multiple common gene variants of small effect size as well as environmental risks. Family-based linkage study findings have not been consistent with a more simplistic ‘major gene effect model’.

### Early candidate gene studies: starting with what we think we know

The earliest studies of ADHD involved testing for association between disorder and variants in candidate genes selected because they were thought likely to be involved in the pathophysiology of ADHD. Multiple candidate gene association studies of ADHD have been published (see Gizer, Ficks, & Waldman, 2009). There is greater confidence in findings that have been replicated and show association in meta-analyses and pooled analyses. For ADHD, a number of these early studies found replicated evidence for association – see Table 1. This relatively large body of replicated findings compared favourably to other research into other psychiatric disorders and further supported evidence that genetic factors were important in the aetiology of ADHD. However, the effect size of each risk variant is small and thus there is no predictive value and nor do they highlight novel biological mechanisms.

**Table 1:** Candidate genes with the most consistent meta-analytic evidence for association with attention deficit hyperactivity disorder

Gene name	Codes for	Variant	Risk allele	Pooled odds ratio
Dopaminergic genes				
*DRD4*	Dopamine D4 receptor	VNTR in exon 3 Polymorphism in promoter region	7-repeat 5-repeat T allele	1.33 (Smith, 2010) 1.68 (Li et al., 2006) 1.21 (Gizer et al., 2009)
*DRD5*	Dopamine D5 receptor	Dinucleotide repeat in 5′ flank	148-bp allele	1.23 (Gizer et al., 2009)
*DAT1*	Carrier protein involved in dopamine reuptake	VNTR in intron 8 Polymorphism in 3′UTR VNTR in 3′UTR	3 repeat G allele 10 repeat	1.25 (Gizer et al., 2009) 1.20 (Gizer et al., 2009) 1.17 (Yang et al., 2007)
Serotonergic genes				
*5HTT*	Carrier protein involved in serotonin reuptake	5HTTLPR polymorphism in promoter region	Long allele	1.31 (Faraone et al., 2005)
*HTR1B*	Serotonin 1B receptor	Polymorphism in exon 1	G allele	1.11 (Gizer et al., 2009)
Other				
*SNAP-25*	Protein involved in neurotransmitter release, synaptic plasticity and axonal growth	Polymorphism in 3′UTR	Not known	1.19 (Faraone et al., 2005)

VNTR, variable number tandem repeat; UTR, untranslated region.

## Searching the whole genome: common and rare variants

### Common DNA sequence variants

One of the rationales for undertaking genetic studies is to discover previously unidentified risk pathways. This has led to ‘hypothesis-free’ whole genome searches for common genetic variants (SNPs) that tagged much (but not all) genetic variation. Detailed accounts of such genome-wide association studies (GWAS) findings and methods are described elsewhere (Franke, Neale, & Faraone, 2009; Stergiakouli & Thapar, 2010). GWAS that have been based on testing common genetic variants (single nucleotide polymorphisms, SNPs) of ADHD have been disappointing. No study to date, including a meta-analysis (Neale et al., 2010), has revealed any gene variant that passes the statistical threshold (*p* = 5 × 10^−8^) for genome-wide significance. The statistical threshold is set high because of the multiple testing burden (recent published studies include testing 500,000–1 million genetic variants; *p*-values of .05 would therefore include 25,000–50,000 SNPs significant by chance). However, it is now apparent that none of these studies was adequately sized. To date, the largest meta-analysis includes fewer than 3000 ADHD cases. Studies of other disorders (e.g. diabetes, schizophrenia) have required thousands and tens of thousands of affected individuals for associated SNPs to reach statistical significance.

There are mixed opinions on the value of such studies. On the one hand, identifying common gene variants that uncover novel biological risk pathways underlying psychiatric disorder is attractive and potentially important. The aim is for this to be achieved by assembling much larger ADHD sample sizes through international collaborations and by performing exome (genetic coding regions) and whole genome sequencing to capture all DNA sequence variation and structural variants (e.g. chromosomal deletions, duplications; Cirulli & Goldstein, 2010; Lander, 2011). Others are more sceptical, suggesting that common genetic variants, at least as currently captured, might not be as important as rare variants in psychiatric disorders (St Clair, 2009), or are worried about the value of identifying risk variants of small effect size (Maher, 2008). The counterarguments, herein, are that the genetic architecture of ADHD appears to be explained by a complex blend of common and rare variants and that the aims of genetic searches are to uncover novel biological and brain risk mechanisms, rather than focus on individual risk factors. All are agreed, however, that better understanding of the pathogenesis of psychiatric disorders, including ADHD, is needed.

### Rare chromosomal structural variants

There has been growing interest in the contribution of rare (traditionally defined as frequency of <1%) rather than common genetic variants to neuropsychiatric disorders, the effects of which have needed to be very large to be detected. It is has long been recognised that rare chromosomal microdeletion syndromes, such as 22q11 microdeletion syndrome (velocardiofacial syndrome, VCFS), that involve a deletion encompassing many genes, can lead to increased risk of ADHD and psychosis (Antshel et al., 2006). Even more subtle chromosomal duplications and deletions, known as structural or copy number variants (CNVs) are becoming increasingly recognised as an important class of genetic variation. All individuals carry CNVs. However, individuals with a variety of brain and neuropsychiatric disorders, including autism (Glessner et al., 2009), intellectual disability (ID; Cooper et al., 2011) and schizophrenia (Grozeva et al., 2012; Raychaudhuri et al., 2010), appear to have an increased burden of rare CNVs, both deletions and duplications, that are of larger size (still submicroscopic, but >500 kb) and encompass multiple different genes.

It is now frequently recommended that clinical genetics services should screen for CNVs in individuals with idiopathic ID (Schaaf, Wiszniewska, & Beaudet, 2011). However, working out which CNVs are pathogenic is not always straightforward. Like with most risk factors, in most instances, there is not a one-to-one relationship between risk and clinical outcome.

The earliest CNV studies of ADHD did not find an increased burden of rare CNVs, but suggested disruption of neurodevelopmental pathways (Elia et al., 2010; Lesch et al., 2011). More recently, the focus has turned to large (>500 kb), rare CNVs. Our UK study found an increased burden of large, rare CNVs in ADHD and that the chromosomal regions involved significantly overlapped with those previously found in autism and schizophrenia (Williams et al., 2010). Although especially high in those with ADHD and ID, the rate of CNVs was not restricted to this group and all analyses were restricted to those without ID. These findings have now been independently replicated (Stergiakouli et al., 2012; Williams et al., 2012). Two specific chromosomal regions have been significantly implicated; one on 16p13.11 that was found in the UK and an Icelandic sample (Williams et al., 2010), and the other potentially involves a region on chromosome 15q11–13 (that contains the nicotinic α7 acetylcholine receptor gene; Stergiakouli et al., 2012; Williams et al., 2012).

As previously highlighted, as would be expected for any risk factor for a complex disorder (e.g. smoking and ischaemic heart disease), the risk factor (herein a higher burden of large rare CNVs) was not found in all children with ADHD and was present in controls (14% vs. 7%; Williams et al., 2010) who remained presumably unaffected (although this was not definitely known as control phenotype data in genotyped samples are not usually available). Large, rare CNVs are interesting because although rare, their effect size has had to be large to be detected [e.g. odds ratio of 13.88 for CNVs spanning 16p13.11 (Williams et al., 2010)], (much larger than is the case for common variants).

Given their rarity, are the children with ADHD who carry large, rare CNVs clinically and biologically atypical? One study found that the children with ADHD with and without such CNVS showed similar clinical features (Langley et al., 2011). Another study showed that the biological pathways impacted upon by large, rare CNVs significantly overlapped with those indexed by common genetic variants (Stergiakouli et al., 2012). These findings suggest that large, rare CNVs do not necessarily result in an atypical form of ADHD. Moreover, the findings suggest that dismissing the contribution of common variants to psychiatric disorder, as some suggest, might be premature. Future research needs to consider models that incorporate both rare and common variants.

Overall, the CNV findings once again suggest that ADHD shows an overlap with other neurodevelopmental disorders, in this instance, autism and schizophrenia. It is of interest that association between CNVs and mood disorders has not been consistently found suggesting that such variants might be especially important in neurodevelopmental disorders (Grozeva et al., 2010).

Many questions remain despite replication. Which of the associated CNVs are causal? Newer research of autism and schizophrenia suggests that both rare sequence and structural variants will be important to examine and that noninherited CNVs that occur *de novo* contribute to sporadic cases. Interestingly, environmental factors might contribute to the appearance of *de novo* mutations (Najmabadi et al., 2011). This has yet to be properly investigated in ADHD. Another critical question is how does the same genetic variant result in a different psychiatric or developmental phenotype? It is not unusual in other areas of medicine for the same risk factor to result in different disorders (e.g. cigarette smoking leads to heightened risks for lung cancer and ischaemic heart disease). Are there additional common and rare genetic and nongenetic factors that shape clinical presentations?

## Antisocial behaviour in ADHD and COMT

ADHD shows considerable clinical heterogeneity in terms of symptom severity, admixture of symptoms, developmental course and comorbidity that are influenced by inherited and noninherited factors. So far there have been few consistent findings that have withstood replication and pooled analyses as to what genetic variants might influence ADHD phenotype variation. One exception is a functional variant in the gene encoding the enzyme COMT (*COMT* val158met) that is the primary mechanism for breakdown of dopamine in the prefrontal cortex. This gene variant modifies COMT enzyme activity whereby those with the val/val genotype have a higher activity version of the enzyme and breakdown dopamine more rapidly. This gene variant has been found to be associated with conduct disorder symptoms in two clinical studies of ADHD (Monuteaux, Biederman, Doyle, Mick, & Faraone, 2009; Thapar et al., 2005), three independent population-based cohorts and a pooled analysis (Caspi et al., 2008; Langley, Heron, O’Donovan, Owen, & Thapar, 2010); although as expected, nonreplications have also been published (Sengupta et al., 2006). The effect size is modest, but strongest for more severe conduct problems [e.g. odds ratio for association with conduct disorder in ADHD vs. no conduct disorder in ADHD = 3.37 (Langley, Heron, et al., 2010)]. Also, the *COMT* val158met variant is only associated with antisocial behaviour in the presence of ADHD and not with antisocial behaviour alone (Caspi et al., 2008; Langley, Heron, et al., 2010). These findings suggest that risk factors for antisocial behaviour in the context of ADHD are not necessarily the same as antisocial behaviour in the general population.

The next question is how might this gene variant exert risk effects? There are a few clues from clinical, cognitive and imaging studies. These studies, that include a meta-analysis, suggest that the *COMT* val158met variant in normal individuals is associated with performance on executive function measures and emotional dysfunction (Mechelli et al., 2009; Meyer-Lindenberg & Weinberger, 2006; Mier, Kirsch, & Meyer-Lindenberg, 2010). A recent study suggests that the *COMT* val158met variant is associated with both executive function and impaired social understanding, but the links with antisocial behaviour in ADHD appear to be mediated through impaired social understanding (Langley, Heron, et al., 2010). If this finding is replicated, and different genetic and environmental risks impact on the same pathways, it suggests alternative treatment targets for the prevention and treatment of antisocial behaviour in ADHD; for example, targeting the social cognitive impairments in ADHD or undertaking pilot studies of COMT inhibitors in those with extreme, intractable aggression.

## What do we know about environmental risks?

Many different environmental factors have been reportedly associated with ADHD, but it has been difficult to identify which are definitely causal (see Lahey, D’Onofrio, & Waldman, 2009; Rutter, 2007; Thapar & Rutter, 2009 for more detailed explanations). As correlation does not necessarily mean causation, extreme caution is needed when interpreting association findings from epidemiological and clinical studies. Many observed associations could arise as a consequence of child and/or parent psychopathology or disposition (e.g. negative mother–child relationship). They could also represent the effects of an unmeasured ‘third variable’ (see Figure 1). Most putative environmental factors (e.g. maternal smoking in pregnancy) are not randomly distributed and could arise because of individuals and/or their parents selecting or shaping their environments. As randomised controlled trials (RCTs) of potential environmental risks and their modification are not always possible or ethical, other types of designs that include quasi experimental, genetically informative and longitudinal studies are helpful in removing some, but not all, of these confounding and reverse causation effects. The aim is to rigorously attempt to disprove causal hypotheses using different designs (see Lahey et al., 2009; Rutter, 2007).

There are many good examples where through such approaches, environmental risks have been found to be causal. For example, for antisocial behaviour, there is consistent evidence from longitudinal (Patterson, DeGarmo, & Forgatch, 2004), randomised control treatment trials (Scott et al., 2010), quasi-experimental research (Costello, Compton, Keeler, & Angold, 2003) and genetically informative studies (Jaffee et al., 2004) that negative parenting, maltreatment and poverty are causal risk factors, especially in those that are also genetically susceptible. For ADHD, this quality of evidence is mainly lacking – see Table 2 for a summary of the environmental factors that have been most widely studied. To limit the scope of this review, we do not focus on environmental factors associated with ADHD continuity or that are manipulated as an intervention for those who already have ADHD (notably dietary interventions).

**Table 2:** Environmental risks that have been most commonly been studied in relation to attention deficit hyperactivity disorder

Pre- and perinatal factors	Environmental toxins	Dietary factors	Psychosocial adversity
Maternal smoking, alcohol and substance misuse *Risk but not proven causal risk factor*	Organophosphate *pesticidesRisk but not proven causal risk factor*	Nutritional deficiencies eg zinc, magnesium, polyunsaturated fatty acids *Correlate not yet proven risk factor*	Family adversity & low income *Correlate not yet proven risk factor*
Maternal stress *Risk but not proven causal risk factor*	Polychlorinated biphenyls *Risk but not proven causal risk factor*	Nutritional surpluses eg sugar, artificial food colourings *Correlate not yet proven risk factor*	Conflict/parent–child hostility *Correlate not yet proven risk factor*

Low birth weight and prematurity *Risk but not proven causal risk factor*	Lead *Risk but not proven causal risk factor*	Low/high IgG foods *Correlate not yet proven risk factor*	Severe early deprivation *Risk, likely causal risk factor*

## Pre and perinatal risks

Maternal smoking during pregnancy is one of the most commonly cited prenatal risks associated with increased rates of ADHD in pooled analyses with an estimated odds ratio of 2.36 (Langley, Rice, van den Bree, & Thapar, 2005). Associations with both ADHD symptoms and diagnoses have been observed using maternal reports of cigarette smoking and objective measures of serum cotinine, whereas a dose–response relationship has also been seen – the more the mother smokes, the greater the associated risk. Similarly, maternal stress during pregnancy has also been noted to be associated with offspring ADHD (e.g. Glover, 2011; Grizenko, Shayan, Polotskaia, Ter-Stepanian, & Joober, 2008). Although there are biologically plausible mechanisms through which these risks could lead to ADHD, it remains unclear whether or not the associations are causal (Thapar & Rutter, 2009). There is growing evidence from studies using genetically sensitive designs, based on children who remain unrelated to their mothers through assisted conception and siblings where the mother has smoked during one pregnancy and not the other (D’Onofrio et al., 2008; Rice, Harold, Boivin, van den Bree, & Thapar, 2009; Thapar et al., 2009), that associations between maternal smoking, stress during pregnancy and ADHD (D’Onofrio et al., 2010; Rice et al., 2009) are due, at least in part, to confounding genetic or other household-level factors. Interestingly, the same designs did not find that inherited confounds contributed to the links between maternal stress and offspring antisocial behaviour and mood/anxiety problems. Also, there is no evidence that as rates of maternal smoking in pregnancy have dropped (Office for National Statistics, 2006), population prevalence rates of ADHD have similarly dropped (Collishaw, Maughan, Natarajan, & Pickles, 2010).

Exposure to other substances during pregnancy has also been found to be associated with ADHD, although with the exception of foetal alcohol syndrome, less robustly. Moderate maternal alcohol use during pregnancy and exposure to illicit substances has been associated with increased risk of ADHD in some, but not all, studies (Linnet et al., 2003).

ADHD, especially the inattentive subtype, has also been found to be associated with low birth weight and prematurity, with a meta-analysis suggesting significant association and an odds ratio of 2.6 for low birth weight (Bhutta, Cleves, Casey, Cradock, & Anand, 2002). Again, it is necessary to consider whether or not these associations are causal and additional study in this area is required as premature birth and low birth weight and obstetric complications might reflect underlying foetal problems (Bailey et al., 1995). However, some genetically informative studies suggest low birth weight is not likely to represent an inherited confound (Groen-Blokhuis, Middeldorp, van Beijsterveldt, & Boomsma, 2011). As is the case for the genetic findings on large, rare CNVs, low birth weight and prematurity are present in only a very small proportion of individuals with ADHD.

Thus, at present, evidence of pre and perinatal factors being causal is lacking. Nevertheless, it is worthwhile having a higher index of suspicion about the presence of ADHD in premature, low birth weight children. Emerging evidence is beginning to challenge the hypotheses that maternal stress and smoking in pregnancy are causal risk factors for ADHD, but further quasi-experimental research is needed.

## Environmental toxins

Exposures to other toxins in prenatal and/or postnatal life have also been considered as increasing the risk of ADHD. In particular, organic pollutants and lead may damage the neural systems implicated in ADHD (Nigg, 2008).

### Pesticides

Organophosphate pesticides have been studied in relation with ADHD, although not widely. A cross-sectional investigation of the relationship between urinary dialkyl phosphate (DAP) metabolites of organophosphates and ADHD diagnosis in middle childhood and early adolescence found that those with detectable urinary dimethyl alkylphosphate (DMAP) metabolite concentrations above the median level had double the odds of ADHD in contrast with those with untraceable levels (Bouchard, Bellinger, Wright, & Weisskopf, 2010). More stringent longitudinal research has found high prenatal organophosphate exposure to be associated with adverse outcomes related to ADHD symptomatology in an investigation of umbilical cord plasma chlorpyrifos levels on subsequent cognitive and motor development in infancy and early childhood (Rauh et al., 2006). However, findings have been inconsistent. Others find that prenatal maternal and postnatal child urinary metabolites of a range of pesticides have no link with ADHD-like symptoms at age 2 years (Eskenazi et al., 2007), but subsequent follow up at 3.5 and 5 years found prenatal DAP metabolites were associated with maternally reported attention difficulties and ADHD symptoms at age 3.5 and these associations became significant at age 5 (Marks et al., 2010). Although commercial use of organochlorine pesticides have now largely been replaced by organophosphates, they do persist in the environment and a Spanish study (Ribas-Fito et al., 2007) did find an association between high cord serum levels of the organochlorine hexachlorobenzene and teacher-rated ADHD behaviours scored by a DSM-IV symptom checklist at age 4.

### Toxic industrial products

Polychlorinated biphenyls (PCBs) are toxic manufactured organochlorine congeners, which were once mass-produced for a variety of industrial and commercial uses and now persist as environmental contaminants. Both human and importantly, animal studies have found that PCB exposure leads to impairments in working memory, response inhibition and cognitive flexibility – neurobehavioural effects, which are comparable with those seen in ADHD (Eubig, Aguiar, & Schantz, 2010). Furthermore, a dose–response relationship, between low level prenatal PCB exposure (as measured by umbilical cord PCB levels) and ADHD-type behaviours in middle childhood was found in a prospective study of a PCB-contaminated area (Sagiv et al., 2010).

### Lead

As with PCBs, human and animal studies of lead exposure have looked at effects on cognitive domains relevant to ADHD; cognitive flexibility, vigilance and alertness have been found to be most dependably affected (Eubig et al., 2010). An association between blood lead level and an actual ADHD diagnosis has been found in several recent studies (including Braun, Kahn, Froehlich, Auinger, & Lanphear, 2006; Froehlich et al., 2009; Nigg, Nikolas, Mark Knowttnerus, et al., 2010).

It is worth noting that there is evidence that both prenatal exposure to PCBs (reviewed in Schantz, Widholm, & Rice, 2003) and even technically ‘low’ blood lead levels (reviewed in Bellinger, 2008a) have more general effects on cognitive function and neurodevelopment as well as on neuropsychological domains specific to ADHD. Nonetheless, due to the neurobiological dysfunction caused by environmental pollutants and the research also implicating them in altered neurodevelopment specific to ADHD, it seems biologically plausible that toxins could indeed have a role. However, as exposure to such substances may not be random, causality cannot yet be firmly inferred from the evidence to date. Conversely, an exception to nonrandom exposure seems to be lead, for which associations with altered neurodevelopment are unlikely to be fully explicable by residual confounding in view of the heterogeneity of settings in which associations have been found (Bellinger, 2008b), and there is now no dietary threshold below which intake can be considered safe (Food and Agriculture Organization of the United Nations and World Health Organization, 2010). At the very least, there is emerging evidence, from both cross-sectional and longitudinal research, of the negative effect of pollutants on neurodevelopment. There needs to be more research making use of quasi-experimental designs and multiple informant assessments of ADHD. Nevertheless, public health and environmental policies aimed at reducing exposure to toxins, particularly for children, is clearly a desirable way forward.

### Diet

Nutritional deficiencies have been studied as aetiological factors, modifying factors and supplementation and exclusion of dietary constituents have also been used as interventions to try and improve outcomes (see Nigg, Lewis, Edinger, & Falk, 2012 for a review). As with environmental toxins, any relationship between diet and ADHD symptoms is unlikely to be straightforward. Although there has been research into several *nutritional deficiencies* as possible aetiological factors in ADHD, (e.g. zinc (reviewed in Arnold & DiSilvestro, 2005), magnesium (Kozielec & Starobrat-Hermelin, 1997) and polyunsaturated fatty acids (Spahis et al., 2008)), there is not strong enough evidence to conclude that such deficiencies have a causal effect. Equally, it is not established whether the biological processes involved in ADHD mean that affected children require an excess of what is considered ‘normal’ ranges of dietary constituents to ameliorate brain function, to justify supplementation in the absence of baseline deficiency. Whilst it is established that extreme nutritional deficiency can interfere with neurodevelopment, the biological effects of more subtle insufficiencies are less clear (Sinn, 2008). Whilst there are also biologically plausible hypotheses as to why diet might be implicated in the development or maintenance of ADHD symptomatology, especially in some individuals, there is not yet consistent enough evidence from research into diet to wholeheartedly support the notion that such factors play a major causal role in ADHD.

## Family adversity and early care-giving

Multiple indicators of psychosocial adversity, including family adversity and low income, have been found to be associated with child mental health problems, including ADHD (Pheula, Rohde, & Schmitz, 2011; Scahill et al., 1999; Taylor & Sonuga-Barke, 2008). Children who suffer maltreatment have also shown a high incidence of ADHD (Famularo, Kinscherff, & Fenton, 1992). A significant difficulty lies in working out the direction of the relationship between adversity and ADHD. For example, family conflict may be seen more commonly in families of a child with ADHD, but is this a cause or consequence of ADHD in the child or parent? Longitudinal and twin designs (Lifford, Harold, & Thapar, 2008, 2009), have suggested that associations between parent–child hostility and ADHD symptoms appear to be accounted for by confounding inherited factors with the exception of the link between mother–son hostility and ADHD symptoms. However, it appears that it is the child’s ADHD symptoms that impact on mother–son hostility, rather than the hostility having a causal role in ADHD. Other studies have shown that treatment with stimulant medication for ADHD improves symptoms, but also improves the mother–child relationship (Schachar, Taylor, Wieselberg, Thorley, & Rutter, 1987). Taken together, this evidence suggests that ADHD symptoms themselves may contribute to family conflict. However, it is possible that family and psychosocial adversity even if it does not play a causal role in the onset of ADHD, may modify its presentation and result in secondary adverse consequences, such as antisocial behaviour (Langley, Fowler et al., 2010). This warrants additional investigation.

ADHD is an early onset disorder. Thus, the contribution of early care-giving requires separate consideration. The impact of severe early deprivation on development of ADHD symptoms has been seen in the English and Romanian Adoptees study (O’Connor & Rutter, 2000; Rutter, Beckett, et al., 2007). This study has the unique strength of being quasi experimental, as it involved examining adopted away children who had been exposed to extreme early deprivation. These results suggest that extreme forms of early deprivation can result in ADHD-type symptoms as well as quasi-autistic symptoms (Rutter, Kreppner, et al., 2007). However, it remains to be seen whether milder forms of early adversity have causal risk effects that could be examined, for example, using adoption study designs. Again, exposures to such risks are likely to be rare and will not account for most ADHD in the population.

At present, with the exception of rare exposure to extreme forms of early adversity, there is no clear-cut evidence that psychosocial adversity causes ADHD, although such factors may well modify its expression and outcomes. Noting adverse circumstances (e.g. maltreatment, poverty) is clearly clinically important with regards to clinical management, but care is needed in ascribing direction of effects, causality and potential blame.

## Gene–environment interplay

As already highlighted, genes and environment do not work independently of each other (see Nigg, Nikolas, & Burt, 2010 for a comprehensive review). Inherited risks can contribute not only directly, but are also likely to operate by increasing the likelihood of exposure to environmental adversity and altering sensitivity to environmental risks and protective factors. There is growing evidence that such interplay is important; for example, gene-environment correlation and interaction play an important role in adolescent depression (Caspi, Hariri, Holmes, Uher, & Moffitt, 2010; Caspi et al., 2003; Thapar, Collishaw, Pine, & Thapar, 2012). Twin and molecular genetic studies have started to test in an exploratory way for gene-environment interaction effects in ADHD (see Nigg, Nikolas, & Burt, 2010). However, as yet, findings have either not been replicated or hypotheses have not been based on neuroscience evidence in the way that was the case for depression. Environmental factors can also impact on early and later development in a more dynamic fashion by influencing how genes are expressed; this is known as epigenetics (see Mill & Petronis, 2008 for a more detailed explanation; Weaver, Diorio, Seckl, Szyf, & Meaney, 2004). This type of complex interplay early on in life could amplify or modify ADHD risk and/or its later consequences and at least in animals such epigenetic effects can be transmitted across generations and reversed.

## Implications for practitioners

### Genetic testing and explaining genetics to families

At present, there are no clinical impacts of the molecular genetic findings. Identified genetic risks on their own have small effect sizes or are very rare and are unlikely to be useful in a predictive or diagnostic sense beyond what can already be predicted by a family history. One exception to where genetic testing might be helpful in the future is for children with intellectual disability who have ADHD and other neurodevelopmental disorders (Cooper et al., 2011; Williams et al., 2010). One of the key aims of molecular genetic studies is to identify underlying, as yet unknown, biological, cognitive, brain and psychosocial risk pathways that lead to the disorder; we provided the example of the *COMT* association with antisocial behaviour in ADHD as possibly being mediated via impaired social understanding.

When families ask about genetic contributions, it is important that they realise familial and genetic risks only increase the probability of ADHD and do not determine the presence of disorder. Heritability estimates refer to populations and do not mean for example that 90% of an individual’s ADHD is caused by genes.

Like other complex disorders, ADHD is not explained by any single risk factor alone and not all those who are exposed to a given risk show disorder. Its occurrence is not explained by inherited factors alone and the pattern of results suggests the contribution of multiple inherited and noninherited factors to its aetiology.

### Conceptualisation of ADHD

In terms of associations with inherited and noninherited factors and adverse consequences, ADHD appears to behave like a trait measure (hyperactivity-impulsiveness and inattentiveness) akin to blood pressure/hypertension; rather than as a qualitatively discrete category. The empirical evidence in favour of using dimensional modifiers in research and practice has been recently highlighted (Willcutt et al., 2012). Evidence of an inherited, molecular genetic and clinical overlap with autism and intellectual disability might strengthen the argument to consider ADHD as a neurodevelopmental disorder, where all such early problems are assessed together in an interdisciplinary health setting with careful assessment of educational and developmental needs and early intervention.

### Considering parents and siblings of the child and integrating services

Given ADHD is familial and heritable, for those seeing children with ADHD, the possibility that a parent/parents might be similarly affected and how this might impact on parenting and the intervention plan (e.g. parenting, family relationships and psycho-educational interventions) needs to be considered (e.g. Johnston, Mash, Miller, & Ninowski, 2012; Thompson et al., 2009). Also, when more than one child in a family has ADHD, there needs to be consideration of the impact on family and parental stress and the practicality of how interventions are delivered.

This could require close working with adult services that will increasingly be required to deal with adult neurodevelopmental problems.

### Implications of genetic findings on treatment choice

Identifying a genetic contribution does not necessarily mean that only biological treatments or medication will be effective. At present, multimodal interventions are recommended for ADHD (NICE, 2008). Moreover, ADHD is not explained by a simple one on one relationship between gene and disorder. Even for single gene disorders where this is the case, for example, phenylketonuria, environmental manipulations (PKU is treated by diet) can be effective.

### Public health implications

At present, there are no clear-cut causal environmental risks that have been identified, with the possible exception of extreme early adversity in some rare instances. There are a number of candidates that require further investigation, but it is critical to use designs that go beyond simply assessing correlations. Nevertheless, there are clear health benefits of mothers not smoking, misusing alcohol and drugs and reducing stress levels in pregnancy, for example, because of causal links with birth weight and prematurity. However, the evidence so far suggests that these risks might not play a direct causal role in ADHD. Similarly, good prenatal and later diet, limiting exposure to toxins is a generally sensible approach. However, if these are not true causal risks, exclusively focusing on them as a means of reducing population rates of ADHD will not be sufficient and could distract from searching for and tackling other potentially modifiable risks.

### Discarding the dichotomisation of genes/biology versus environment in practice as well as research

Finally, a key message of this review for practitioners and families is that there is overwhelming evidence that the polarisation between genetic/biological and environmental factors, perpetuated by some due to misinterpretation of aetiological and genetics research in relation to complex disorders, is incorrect and unhelpful. Indeed, they are complementary rather than competing explanations.

**Key points**

**Key practitioner message:**

- There is no single risk factor that explains ADHD. Many different risks and risk pathways may all lead to the same clinical phenotype.
- Both inherited and noninherited factors contribute to ADHD, and their effects are interdependent.
- The most consistently found risk factors for ADHD include having a relative with the disorder, large rare CNVs, candidate gene variants, extreme early adversity, pre and postnatal exposure to lead and low birth weight/prematurity.
- The risk factors implicated in ADHD tend to have small effect sizes or are rare. They also may increase risk of many other types of psychopathology.
- It is important to remember that associations between ADHD and investigated risk factors may not necessarily be due to causal effects.
- There is important overlap with other neurodevelopmental problems, including autistic spectrum disorders, as well as comorbidity with other psychiatric and behavioural disorders.

**Areas for future research:**

- Further genetic studies, that are adequately sized, may be useful in allowing DNA sequence variation and structural variants for ADHD to be captured.
- Future studies into environmental risk factors associated with ADHD need to use designs that go beyond assessing correlation and look for evidence of causal links.
- For children with ADHD, the possibility that a parent might be similarly affected needs to be considered, and studies looking at how this may impact on the intervention and outcome for the child, could provide important information for future management.
